# Fermented Wheat Germ Extract as a Redox Modulator: Alleviating Endotoxin-Triggered Oxidative Stress in Primary Cultured Rat Hepatocytes

**DOI:** 10.1155/2020/3181202

**Published:** 2020-12-08

**Authors:** Máté Mackei, Júlia Vörösházi, Csilla Sebők, Zsuzsanna Neogrády, Gábor Mátis, Ákos Jerzsele

**Affiliations:** ^1^Division of Biochemistry, Department of Physiology and Biochemistry, University of Veterinary Medicine, István utca 2, H-1078 Budapest, Hungary; ^2^Department of Pharmacology and Toxicology, University of Veterinary Medicine, István utca 2, H-1078 Budapest, Hungary

## Abstract

Bioactive compounds such as benzoquinone derivates presented in fermented wheat germ extract (FWGE) have several positive effects on overall health status of humans and animals alike. Since available data regarding the antioxidant activity of FWGE are limited, the aim of our study was to investigate its effects on the cellular redox homeostasis applying primary hepatocyte cell cultures of rat origin. Cultures were challenged to lipopolysaccharide (LPS) treatment for 2 or 8 hours to trigger inflammatory response. Further, culture media were concomitantly supplemented with or without FWGE (Immunovet®, 0.1% and 1%). In order to monitor the metabolic activity of the cell cultures, CCK-8 test was applied, while reactive oxygen species (ROS) production was measured using Amplex Red method. Malondialdehyde concentration of culture media as a specific marker of lipid peroxidation and the activity of glutathione peroxidase in cell lysates were also determined to monitor the redox status of the cultures. Based on our findings, it can be concluded that FWGE did not show cytotoxic effects in any applied concentration in cell cultures. Furthermore, FWGE efficiently decreased cellular ROS production and lipid peroxidation rate in case of LPS-induced inflammatory response. However, without LPS treatment, higher concentration of FWGE increased the rate of both ROS and malondialdehyde synthesis. This observation may refer to the prooxidant activity of high dose FWGE, which is an important beneficial effect regarding tumor cells. However, in case of noninflamed hepatocytes, considering the results of glutathione peroxidase activity, the application of the product did not result in severe oxidative distress. In accordance with the abovementioned findings, FWGE as a redox modulator, applied in the appropriate concentration, can serve as a promising candidate in the supplementary therapy of patients suffering from various inflammatory diseases, decreasing the free radical generation, thus avoiding the occurrence of cytotoxic effects.

## 1. Introduction

Based on its various beneficial biological effects, fermented wheat germ extract (FWGE) is successfully used in human medicine, mainly in the supportive therapy of people suffering from cancer. Bioactive compounds—most importantly different benzoquinone derivates—found in FWGE provide significant anticancer effects by influencing several cellular molecular mechanisms [1]. The FWGE stimulates the immune response against tumor cells by decreasing the MHC-I expression in the cell membrane and rendering cancer cells more effectively be recognized by natural killer (NK) cells [1]. In addition, FWGE increases tumor necrosis factor *α* (TNF*α*) production by macrophages, leading to improved immune response towards tumor cells, inhibition of angiogenesis, and increased apoptosis of the target cells [2]. Furthermore, FWGE is also able to increase interleukin 1*α* (IL-1*α*), IL-2, IL-5, and IL-6 levels [3], which are considered to be among the main regulatory molecules of the inflammatory response. Beyond its immunomodulatory effects, FWGE can enhance oxidative stress in tumor cells, inducing cell destruction caused by the produced free radicals [4]. Moreover, it has the ability to affect the carbohydrate and nucleotide metabolism of cancer cells. As an example, by the inhibition of hexokinase enzyme, it is able to decrease cellular ATP production and hinder the synthesis of pentoses, which are necessary for cell division [5]. Besides, FWGE impedes the activity of ribonucleotide reductase enzyme, directly decelerating the production of nucleotides needed for the DNA synthesis [6]. As a result of all the mentioned effects, FWGE is able to effectively decrease the proliferation of several malignant tumor types and to increase the apoptosis of these cells. These findings were initially confirmed in studies on HT-29 colorectal adenocarcinoma and HL-60 leukaemia cell lines [2]. By virtue of the efficient antitumor activity of FWGE, slower tumor growth rate has been detected, resulting in longer life expectancy. The FWGE-triggered improvement of the general health condition and the successful prevention of cancer-associated cachexia can also contribute to the better prognosis [7]. The decreased velocity of tumor growth and metastasis formation was described in case of numerous forms of tumors, such as in melanoma, in neuroblastoma, and in different cervical, testicular, or thyroid cancer types [6].

With the modulation of cellular and humoral immune response, FWGE can serve as a considerable effector not just in point of its immunomodulatory activity towards the neoplastic cells but also as a result of its general immunostimulatory effects [6]. Significant enhancement of the immune response was detected in FWGE treated, beforehand immunosuppressed mice, mainly resulting from the effective induction of differentiation and blast transformation of lymphocytes [8]. Beyond these results, FWGE is capable to be used in different immune-mediated diseases and to resolve the immunosuppressive effects caused by cyclophosphamide treatment [6, 9]. Considerable anti-inflammatory activity of FWGE was also detected based on the inhibition of cyclooxygenase (COX) enzymes, successfully supporting the action of nonsteroidal anti-inflammatory drugs (NSAIDs) [7].

Regarding the potential antioxidant effects of FWGE, containing high concentration of bioactive free radical scavenger molecules, only limited data are available. It was reported to decrease the amount of reactive oxygen species (ROS) such as superoxide anion radicals [10]. However, further research is required concerning the antioxidant activity of FWGE.

Following its application in human medicine, FWGE was also introduced to veterinary practice for companion animals, and based on its immunostimulatory, anti-inflammatory, and suggested antioxidant effects, it can serve as a proper candidate for maintaining and improving the general health status of the patients [11]. The application of FWGE can be of high importance in case of elderly, debilitated animals, suffering in various chronic diseases [12]. Furthermore, applying FWGE in companion animals affected by neoplastic diseases may also be promising, based on its antitumor activity and its ability to improve general health condition. In addition, FWGE can be also effectively used as a natural growth promoter in chicken [11] and turkey [12], contributing to improved productivity and health conditions of farm animals. In accordance with its antimicrobial activity, FWGE is proved to be efficient to treat mycoplasma infection [13] and to mitigate the spreading of Salmonella Typhimurium in chicken; further, the efficiency of different applied vaccines can be also enhanced by dint of FWGE's immunostimulatory effects [14].

In spite of the above described effects—such as antitumor, immunostimulatory, anti-inflammatory, and antimicrobial activity—only limited data are available about the possible effects of FWGE on the antioxidant status of eukaryotic cells. Hence, in this present study, we aimed to investigate the effects of FWGE (Immunovet®) on the redox homeostasis as well as on the oxidative status of the liver. The investigations were carried out using primary hepatocyte cultures of rat origin, which model can be a proper tool to observe the exact molecular mechanisms on the cellular level. This *in vitro* study—applying rat as a widely used and accepted model animal in the research—can serve with relevant and valuable information about FWGE-induced alterations in farm and companion animals, moreover in humans.

## 2. Materials and Methods

All reagents used in the study were purchased from Sigma-Aldrich (Darmstadt, Germany), except when otherwise specified. Animal procedures described hereinafter were performed in strict accordance with the national and international law along with institutional guidelines and were confirmed by the Local Animal Welfare Committee of the University of Veterinary Medicine, Budapest, and by the Government Office of Pest County, Food Chain Safety, Plant Protection, and Soil Conservation Directorate, Budapest, Hungary.

### 2.1. Cell Isolation and Culturing Conditions

Isolation and culturing of primary rat hepatocytes were carried out based on our formerly developed and published method [15]. Briefly, hepatocyte isolation was performed using 8-week-old Wistar rats (approx. 200-250 g). Animals were kept and fed according to the actual Hungarian and European animal welfare laws. After carbon dioxide narcosis, median laparotomy was performed followed by the cannulation of the vena portae and the thoracic section of the vena cava caudalis. The liver was flushed and exsanguinated through the portal system, using different buffers and multistep perfusion. In order to recirculate the buffers, the effusing amount of the solutions was collected via the vena cava caudalis.

To perfuse the liver, 300 mL ethylene glycol tetraacetic acid (EGTA, 0.5 mM) containing Hanks' Balanced Salt Solution (HBSS) buffer, 200 mL EGTA-free HBSS buffer, and finally, 130 mL EGTA-free HBSS buffer, supplemented with 50 mg type IV collagenase (Serva, Duisburg, Germany), and 2.5 mM CaCl_2_ and MgCl_2_ were used.

During the liver perfusion, all of the applied buffers were warmed up to 40°C and oxygenated with Carbogen (95% O_2_, 5% CO_2_); the velocity was set to 30 mL/min. The collagenase containing buffer was recirculated until the complete disintegration of the liver parenchyma. After excision of the liver and disruption of the capsule, cell suspension was filtered using sterile gauze sheets. Cell suspension was placed for 50 min into 25 mg/mL bovine serum albumin (BSA) containing ice-cold HBSS in order to avoid undesired cluster formation.

Hepatocytes were isolated using low speed multistep differential centrifugation (3 times, 100 × g, 2 min), and the gained pellets were resuspended in Williams' Medium E supplemented with 50 mg/mL gentamycin, 2 mM glutamine, 20 IU/L insulin, 4 *μ*g/L dexamethasone, 0.22% NaHCO_3_, and in the first 24 h of culturing with 5% foetal bovine serum (FBS).

**Figure 1:**
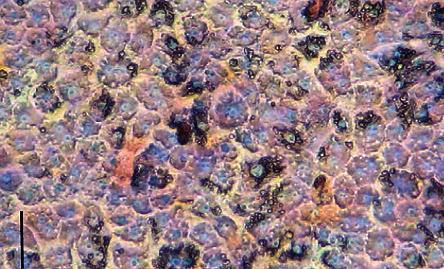
Primary hepatocyte cultures of rat origin after 24 h incubation. Giemsa staining, bar = 30 *μ*m.

After resuspendation, viability of hepatocytes was tested by trypan blue exclusion test, always exceeding 90%. The number of the cells was determined by cell counting in Bürker's chamber to further adjust the appropriate cell concentrations to 10^6^ cells/mL. Hepatocytes were seeded onto 96- and 6-well Greiner Advanced TC cell culture dishes (Greiner Bio-One Hungary Kft., Mosonmagyaróvár, Hungary) previously coated with collagen type I (10 *μ*g/cm2), using 200 *μ*L/well seeding volume in the 96-well plates and 2 mL/well in the 6-well plates. Cultures were incubated at 37°C and 100% relative air humidity. Cell culture media were changed after 4 h, and confluent monolayer cell cultures were gained after 24 h incubation (Figure 1).

### 2.2. Treatments of Cultured Cells

After 24 h, culturing cells were treated using cell culture media supplemented with 0 (control) or 10 *μ*g/mL Salmonella enterica serovar. Typhimurium derived lipopolysaccharide (LPS) for 2 and 8 h incubation time. Further, in both of the control and LPS-challenged cultures, subgroups were prepared using 0.1% and 1% FWGE prepared from Immunovet®, silymarin (50 *μ*g/mL), or ursodeoxycholic acid (UDCA, 200 *μ*g/mL) containing cell culture medium. In the latter two cases, cultures were treated with proved hepatoprotective and antioxidant substances.

To gain the FWGE working solutions (Immunovet®), 1 g of FWGE granules was homogenized using a mortar until a fine powder was received and dissolved in 10 mL sterile phosphate buffered saline (PBS) solution. The gained stock solution (100 mg/mL; 10%) was filtered in different steps, using gauze sheets (3 layers, 2 times filtering), a cell strainer (70 *μ*m pore size), and a sterile filter (0.22 *μ*m pore size) in the end (Merck Millipore, Burlington, MA, USA). Stock solution (10%) was diluted with PBS to 1% and 0.1% concentrations.

On both of the 96- and 6-well plates, 6 replicates were prepared per one treatment group (*n* = 6). In case of the 6-well plates, following either 2 or 8 h incubation, samples were taken from the cell culture media. Thereafter, wells were washed with PBS, and cells were lysed using Mammalian Protein Extraction Reagent (M-PER™, Thermo Fisher Scientific, Waltham, MA, USA). All of the collected samples were stored until further analysis at −80°C.

### 2.3. Measurements of Cellular Metabolic Activity, Extracellular H_2_O_2_ and Malondialdehyde Concentrations, and Glutathione Peroxidase Activity

Following the treatments, metabolic activity of cells cultured on 96-well plates was checked using CCK-8 assay (Dojindo, Rockville, USA), monitoring the total amount of NADH+H^+^ produced in the cellular catabolic reactions, successfully reflecting also to the potential cytotoxic effects. According to the manufacturer's instructions, 10 *μ*L CCK-8 reagent and 100 *μ*L Williams' Medium E were added to the cultured cells, and after 2 h of incubation at 37°C, the absorbance was measured at 450 nm with a Multiskan GO 3.2 reader (Thermo Fisher Scientific, Waltham, MA, USA).

Extracellular H_2_O_2_ concentration was detected in the culture medium using the fluorimetric Amplex Red method (Thermo Fisher Scientific, Waltham, MA, USA). After 30 min incubation of 50 *μ*L freshly prepared, Amplex Red (100 *μ*M) and HRP (0.2 U/mL) containing working solution with 50 *μ*L culture medium at room temperature (21°C), fluorescence (*λ*ex = 560 nm; *λ*em = 590 nm) was detected using a Victor X2 2030 fluorometer (Perkin Elmer, Waltham, MA, USA).

Malondialdehyde (MDA) concentration as a marker of lipid peroxidation was monitored in cell culture media with a specific colorimetric test. According to the protocol, 300 *μ*L freshly prepared thiobarbituric acid (TBA) stock solution was mixed with 100 *μ*L cell culture media. Solutions were incubated at 95°C for 1 h followed by 10 min cooling on ice. Absorbance was measured at 532 nm with a Multiskan GO 3.2 reader (Thermo Fisher Scientific, Waltham, MA, USA).

As one of the most prominent members of the antioxidant defence system, activity of glutathione peroxidase enzyme of the cell lysates was also determined using a colorimetric kinetic assay. At first, GPx Assay Buffer was prepared according to the manufacturer's protocol, and 455 *μ*L was mixed with 25 *μ*L of NADPH Assay Reagent and 5 *μ*L substrate solution (tert-butyl hyperoxide). The decrease of absorbance was continuously detected at 340 nm (initial delay: 15 sec; interval: 10 sec; number of readings: 6). Enzyme activity was calculated using the formula provided by the manufacturer.

### 2.4. Statistics

All the data analysis was performed using the R 3.5.3. software (GNU General Public License, Free Software Foundation, Boston, MA, USA). On both of 96- and 6-well plates, six wells were included in one treatment group. Normal distribution and homogeneity of variance were checked by Shapiro-Wilk test and Levene's test, respectively. Differences between various groups were assessed using one-way analysis of variance (ANOVA) and Tukey's post hoc tests for pairwise comparisons. Results were assessed as the mean ± standard error of the mean (SEM). Differences were assumed significant at *P* < 0.05. Results of the FWGE, silymarin, and UDCA treated groups were compared to the respective control groups (LPS free or LPS supplemented control groups). The effects of LPS supplementation were considered as main effect compared to the control groups without LPS treatment.

## 3. Results

### 3.1. Measurement of Cellular Metabolic Activity

**Figure 2:**
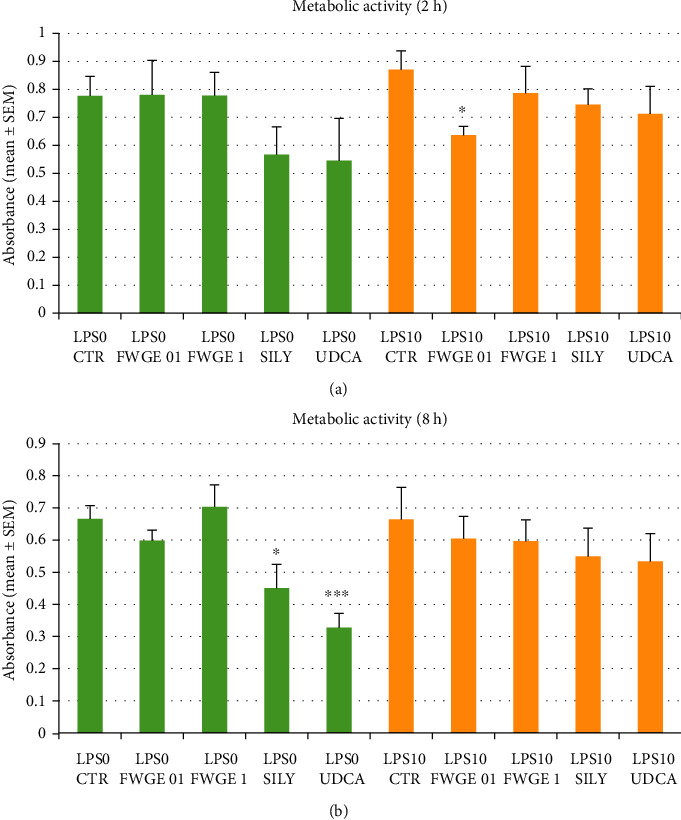
Metabolic activity of cultured cells after 2 h (a) and 8 h (b) incubation measured with the CCK-8 assay. LPS 0: cultures with no LPS exposure; LPS 10: LPS treated cultures (10 *μ*g/mL); FWGE 01: 0.1% FWGE; FWGE 1: 1% FWGE; SILY: silymarin (50 *μ*g/mL); UDCA: ursodeoxycholic acid (200 *μ*g/mL). Mean ± SEM, ^∗^*P* < 0.05, ^∗∗∗^*P* < 0.001.

Metabolic activity of the cultured cells was monitored using CCK-8 assay, indicating the cellular aerobe catabolic processes. According to our results, the majority of the applied treatments were not able to affect the metabolic activity of the cultures, except the observed significant decrease in case of the 2 h long 0.1% FWGE (*P* = 0.016; Figure 2(a)) exposure in the LPS challenged groups and the 8 h long silymarin and UDCA (*P* = 0.029 and *P* < 0.001; Figure 2(b)) treatments of LPS free control cells.

### 3.2. Measurement of H_2_O_2_ Production

**Figure 3:**
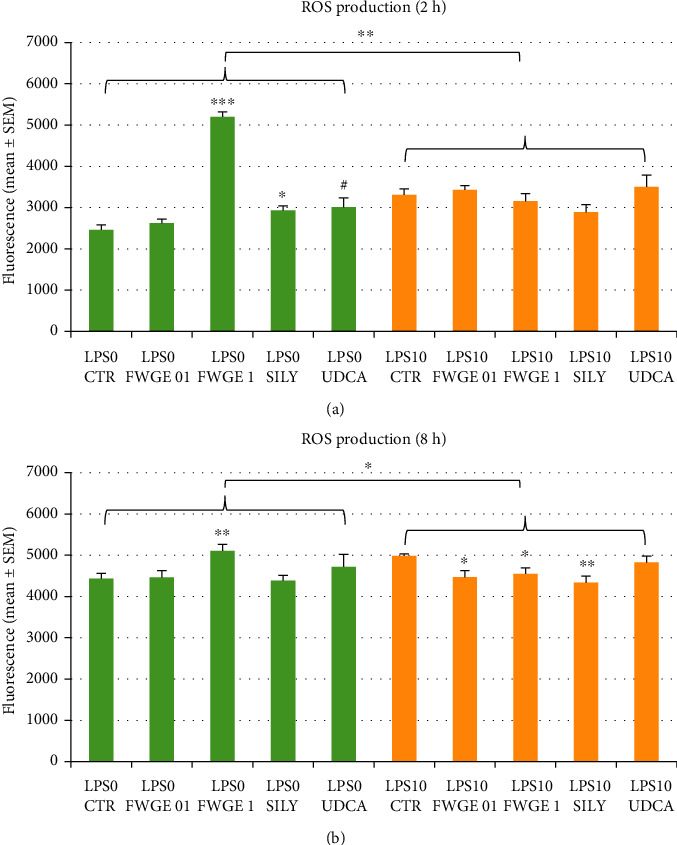
Extracellular ROS production of cell cultures after 2 h (a) and 8 h (b) incubation measured with the Amplex red method. LPS 0: cultures with no LPS exposure; LPS 10: LPS treated cultures (10 *μ*g/mL); FWGE 01: 0.1% FWGE; FWGE 1: 1% FWGE; SILY: silymarin (50 *μ*g/mL); UDCA: ursodeoxycholic acid (200 *μ*g/mL). Mean ± SEM, #*P* < 0.10, ^∗^*P* < 0.05, ^∗∗^*P* < 0.01, ^∗∗∗^*P* < 0.001.

The extracellular ROS production of the cells (H_2_O_2_ concentration in the cell culture media) was monitored with the Amplex Red method. ROS concentrations were elevated after both of the incubation times in the LPS treated groups (2 h incubation: *P* = 0.0012; 8 h incubation: *P* = 0.036; Figures 3(a) and 3(b)).

In cell cultures without LPS treatment, FWGE applied in 1% significantly increased ROS concentration of the cell culture media after both 2 h and 8 h incubation (*P* < 0.001 and *P* = 0.007, Figures 3(a) and 3(b)). Similarly to these findings, ROS production was significantly and tendentiously increased as a result of 2 h silymarin and UDCA treatments without LPS application (silymarin: *P* = 0.014; UDCA: *P* = 0.058; Figure 3(a)).

On the other hand, LPS triggered elevation of the ROS levels was significantly decreased applying both FWGE and silymarin supplementation after 8 h incubation (0.1% FWGE: *P* = 0.020; 1% FWGE: *P* = 0.027; silymarin: *P* = 0.006; Figure 3(b)).

### 3.3. Determination of Malondialdehyde Concentration

**Figure 4:**
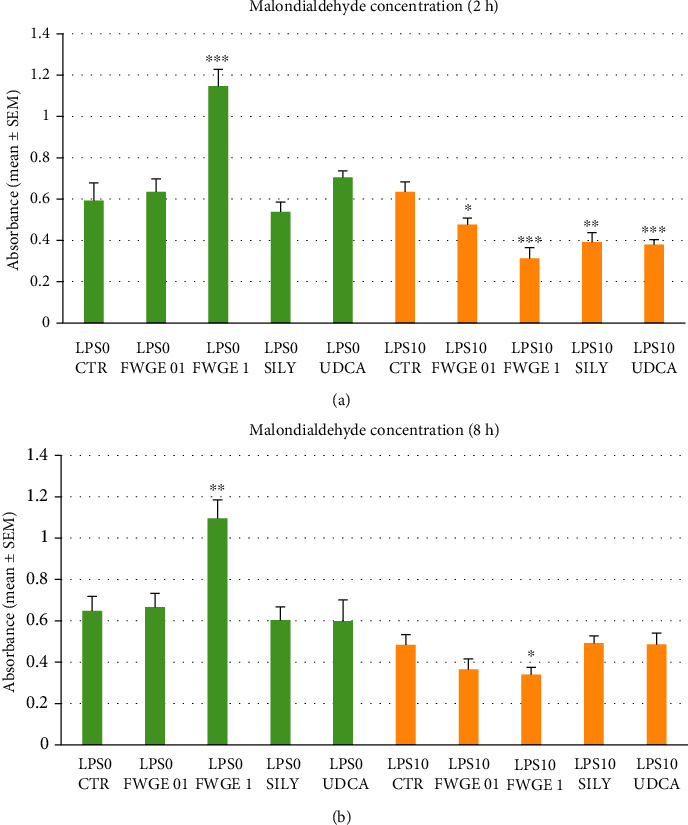
Malondialdehyde concentration measured in the cell culture media after 2 h (a) and 8 h (b) incubation. LPS 0: cultures with no LPS exposure; LPS 10: LPS treated cultures (10 *μ*g/mL); FWGE 01: 0.1% FWGE; FWGE 1: 1% FWGE; SILY: silymarin (50 *μ*g/mL); UDCA: ursodeoxycholic acid (200 *μ*g/mL). Mean ± SEM, ^∗^*P* < 0.05, ^∗∗^*P* < 0.01, ^∗∗∗^*P* < 0.001.

In order to monitor the lipid peroxidation processes in the cell cultures, MDA concentration was measured in the media after both incubation times. In the cells with no LPS treatment, FWGE applied in 1% concentration caused significantly higher MDA level after 2 h as well as 8 h incubation time (2 h: *P* < 0.001; 8 h: *P* = 0.003; Figures 4(a) and 4(b)). However, together with LPS treatment, 1% FWGE significantly decreased the production of MDA after both incubation times (2 h: *P* < 0.001; 8 h: *P* = 0.044; Figures 4(a) and 4(b)). Similarly to these findings, following 2 h of LPS exposure, FWGE applied in lower concentration as well as silymarin and UDCA treatment significantly decreased the MDA concentration of the cell culture supernatants (0.1% FWGE: *P* = 0.018, silymarin: *P* = 0.004, UDCA: *P* < 0.001; Figure 4(a)).

### 3.4. Measurement of Glutathione Peroxidase Activity

**Figure 5:**
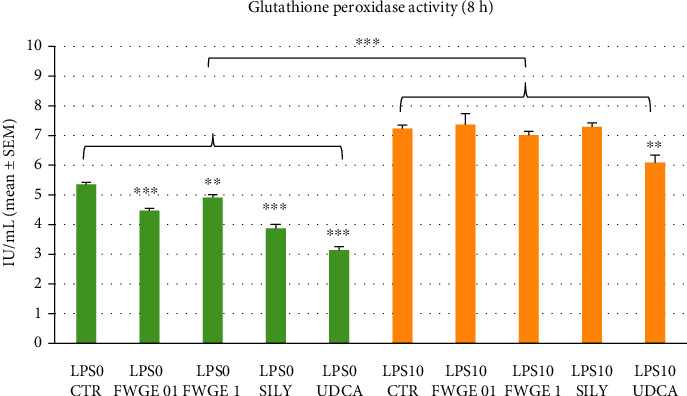
Glutathione peroxidase activity of the cultured cells after 8 h incubation. LPS 0: cultures with no LPS exposure; LPS 10: LPS treated cultures (10 *μ*g/mL); FWGE 01: 0.1% FWGE; FWGE 1: 1% FWGE; SILY: silymarin (50 *μ*g/mL); UDCA: ursodeoxycholic acid (200 *μ*g/mL). Mean ± SEM, ^∗∗^*P* < 0.01, ^∗∗∗^*P* < 0.001.

The activity of glutathione peroxidase enzyme of the lysed cells was monitored after 8 h incubation. As the effect of LPS challenge, enzyme activity was significantly elevated (*P* < 0.001; Figure 5.). Both of the applied FWGE concentrations along with the silymarin and UDCA incubation decreased the activity of glutathione peroxidase in the cell cultures without LPS treatment (0.1% FWGE, silymarin, and UDCA: *P* < 0.001; 1% FWGE: *P* = 0.004; Figure 5). In LPS exposed cells, only the UDCA treatment was able to decrease the activity of glutathione peroxidase enzyme in a significant manner (*P* = 0.002; Figure 5).

## 4. Discussion

In the present study, cellular effects of FWGE applied at different concentrations were investigated in cultured primary rat hepatocytes. FWGE is a standardized extract fermented by *Saccharomyces cerevisiae*, adjusted to a yield of 0.4 mg/g 2,6-dimethoxy-p-benzoquinone. The impact of FWGE on metabolic activity and redox homeostasis of cell cultures was monitored and analyzed in comparison with the potent hepatoprotective agents silymarin and UDCA. Applied concentrations of FWGE were set up based on the available literature data. In other studies, 1% FWGE (10 mg/mL) showed cytotoxic and cytostatic activity after 24 h incubation, while in case of pancreas and mammary tumors, it had cytotoxic effects already at 0.5% [4]. Further, higher sensitivity of tumorigenic cells does not necessarily correspond with the potential cytotoxic effects of FWGE in healthy, nontumorigenic cell types. However, considering the relatively higher vulnerability of primary hepatocyte cultures towards different toxic effects, in our study, FWGE was applied at 0.1% and 1% (1 and 10 mg/mL) concentrations. Dose of LPS (10 *μ*g/mL) was determined previously in our studies regarding inflammatory processes using primary hepatocyte models [16], mimicking a severe inflammatory response. Incubation times (2 and 8 h) were also chosen in accordance with the similar investigations carried out in hepatocyte cell cultures, mimicking an acute and a subacute hepatic inflammation.

Cellular metabolic activity was monitored with the CCK-8 method. With the help of the assay, information can be gained regarding aerobe catabolic processes of biological oxidation by screening cellular NADH+H+ production. Based on our results, in case of LPS challenged cell cultures, a slightly but significantly decreased catabolic activity was detected in the samples treated with 0.1% FWGE (Figure 2(a)). This slight decrease presumably does not mean an intense cytotoxic effect but can refer to a rearrangement in the activity of different metabolic processes. Therefore, according to our findings, FWGE did not cause cytotoxic effects at 0.1 and 1% concentrations after 2 and 8 h incubation time in hepatocytes under normal physiological circumstances.

ROS production, as a key indicator of oxidative stress, was monitored by the determination of H_2_O_2_ concentration of the cell culture media. ROS production of LPS nontreated cultures was significantly elevated as the effect of 1% FWGE application after both 2 h and 8 h incubation (Figures 3(a) and 3(b)). This finding suggests that the application of FWGE at elevated concentrations may lead to oxidative stress in healthy hepatocytes not affected by inflammation. In contrast to these effects, in case of LPS challenged cultures, similarly to silymarin treatment, FWGE was capable to decrease the intensity of oxidative stress (Figure 3(b)). For this reason, based on our results, it can be stated that FWGE can show different effects on ROS production of healthy cells and cultures affected by severe inflammatory processes. Presumably, in the first scenario, it may have a mild prooxidant activity at high doses, while in the latter case, it can serve with relevant antioxidant effects. Depending on the applied concentration and other different factors, numerous investigations can be found in the literature referring the possibility of prooxidant effects of molecules considered as antioxidants. As an example, both pro- and antioxidant activities of flavonoids [17] or pyrroloquinoline quinone, applied widely as farm animal feed additive and redox modulator [18], were described under *in vitro* as well as *in vivo* conditions.

H_2_O_2_, investigated in our study, plays a multifaceted role in the regulation of the cellular redox homeostasis [19]. According to our recent knowledge, similarly to Ca^2+^, ATP, or cAMP, H_2_O_2_ belongs to the group of main secondary messenger molecules [20]. Its increased concentration can lead to alterations of the cellular morphology and metabolism, decreasing or increasing cell proliferation; further, it is also involved in the activation of immune cells. It is of great importance that normal, well-regulated production of H_2_O_2_ is necessary for the development and maintenance of the so called oxidative eustress, resulting in beneficial physiological effects and efficacious cellular adaptation to different external factors [19]. By this reason, elevated intensity of ROS production does not necessarily mean pathologic alterations of the cells as long as it is occurring within the confines of appropriately regulated and beneficial oxidative eustress. The presence of prooxidants can however contribute to the occurrence of oxidative distress, which can drive to cellular impairments such as the damage of DNA and proteins together with increased intensity of lipid peroxidation [19]. It is also an important aspect that the antitumor activity of FWGE is partially based on the prooxidant effect of the extract, since the increased H_2_O_2_ production and the therefore occurring oxidative distress entails the destruction of the cancer cells, resulting in slower growth and smaller tumor size [4].

Concerning the results of our study, FWGE administration in higher dose can supposedly increase ROS production of healthy liver cells; however, in case of inflammatory processes, application for a longer time, both in lower and higher concentration, is able to decrease H_2_O_2_ production of cells, restoring the conditions of normal oxidative eustress. The presented data reminds to the importance of appropriate therapeutic dosage, avoiding the application of the antioxidants in too high concentration leading to prooxidant activity in healthy organisms.

One of the main consequences of the oxidative stress can be the increased cellular lipid peroxidation and the subsequent impairment of the cell membranes, resulting in the increment of membrane permeability. Lipid peroxidation was monitored by measuring the MDA concentration of cell culture media, which indicates the status of the cellular membranes. The FWGE administered in higher dose (1%) significantly increased MDA concentration, similarly to ROS production, in the LPS nontreated cultures mimicking healthy conditions after both incubation times (Figures 4(a) and 4(b)). In contrast, elevated MDA production in LPS triggered inflammation, in correlation with ROS production, was efficiently decreased when applying both FWGE concentrations after 2 h incubation and by 1% FWGE extract after 8 h incubation (Figures 4(a) and 4(b)). These results together with ROS production refer to the significance of appropriately chosen concentration of FWGE in order to avoid the adventitiously occurring prooxidant effects in normal, healthy circumstances. However, accordingly applied FWGE can serve as proper tool not only to remarkably decrease cellular free radical production but also to avoid the possible membrane damage caused by intense lipid peroxidation.

The function of the whole glutathione system was monitored with the applied glutathione peroxidase assay by assessing the NADPH+H^+^ amount needed for converting glutathione back to its reduced form by glutathione reductase. Glutathione is one of the most prominent antioxidants, oxidized by glutathione peroxidase enzyme, while taking part in the binding and inactivating of generated free radicals. With our measurements, we were able to achieve a more accurate picture regarding the function of the whole glutathione system, which—as a crucial intracellular antioxidant—refers to the oxidative status of the cells.

According to the present results, LPS treatment significantly increased the activity of the glutathione peroxidase (Figure 5), reflecting the more intense function and capacity of this antioxidant defence system due to the LPS provoked oxidative distress. Further, other studies showed similar results regarding the enhanced glutathione peroxidase enzyme activity observed in the liver of LPS challenged mice [21]. Similarly to silymarin and UDCA, FWGE was able to significantly reduce the activity of glutathione defence system in LPS nontreated cell cultures. The mildly decreased activity of the glutathione peroxidase caused by FWGE, indicating the overall oxidative status of the cells, suggests that the observed elevated ROS and MDA levels do not necessarily contribute to oxidative distress in noninflamed hepatocytes. In order to clarify possible species-specific differences, further investigations using cell culture models of companion animal origin would be also beneficial in the future to observe the effects of FWGE in these target species.

## 5. Conclusions

Summarizing our results, it can be stated that the extract of FWGE containing Immunovet® product applied in well considered appropriate concentration did not possess cytotoxic effect in rat-derived primary hepatocyte cultures. The FWGE effectively decreased the ROS production of cultured cells and the consecutively occurring lipid peroxidation in case of LPS triggered inflammation in the applied *in vitro* hepatic model. Notwithstanding that the incidental prooxidant activity of FWGE in higher concentration is also remindful, it does not necessarily lead to oxidative distress. In conclusion, FWGE as a redox modulator can provide good possibilities in alleviating inflammation associated oxidative distress, preventing cell destruction and hence improving general health condition.

## Data Availability

All relevant data analyzed during the current study are available from the corresponding author (mackei.mate@univet.hu) on request.

## References

[1] Iyer A., Brown L. (2011). Fermented wheat germ extract (Avemar) in the treatment of cardiac remodeling and metabolic symptoms in rats. *Evidence-based Complementary and Alternative Medicine*.

[2] Mueller T., Voigt W. (2011). Fermented wheat germ extract - nutritional supplement or anticancer drug?. *Nutrition Journal*.

[3] Telekes A., Hegedűs M., Chae C.-H., Vékey K. (2009). Avemar (wheat germ extract) in cancer prevention and treatment. *Nutrition and Cancer*.

[4] Otto C., Hahlbrock T., Eich K. (2016). Antiproliferative and antimetabolic effects behind the anticancer property of fermented wheat germ extract. *BMC Complementary and Alternative Medicine*.

[5] Boros L. G., Lapis K., Szende B. (2001). Wheat germ extract decreases glucose uptake and RNA ribose formation but increases fatty acid synthesis in MIA pancreatic adenocarcinoma cells. *Pancreas*.

[6] Boros L. G., Nichelatti M., Shoenfeld Y. (2005). Fermented wheat germ extract (Avemar) in the treatment of cancer and autoimmune diseases. *Annals of the New York Academy of Sciences*.

[7] Telekes A., Resetar A., Balint G. (2007). Fermented wheat germ extract (Avemar) inhibits adjuvant arthritis. *Annals of the New York Academy of Sciences*.

[8] Patel S. (2014). Fermented wheat germ extract: a dietary supplement with anticancer efficacy. *Nutritional Therapy & Metabolism*.

[9] Ehrenfeld M., Blank M., Shoenfeld Y., Hidvegi M. (2016). AVEMAR (a new benzoquinone-containing natural product) administration interferes with the Th2 response in experimental SLE and promotes amelioration of the disease. *Lupus*.

[10] Hidvégi M., Rásó E., Tömösközi-Farkas R. (1999). MSC, a new benzoquinone-containing natural product with antimetastatic effect. *Cancer Biotherapy and Radiopharmaceuticals*.

[11] Jerzsele Á., Somogyi Z., Szalai M., Kovács D. A. (2020). Fermentált búzacsíra-kivonat hatása brojlercsirkék mesterséges Salmonella Typhimurium fertőzésére.

[12] Kósa E., Nagy G., Jakab L., Hidvégi M., Resetár Á., Sári I. The effect of Immunovet-HBM® supplement on broiler turkey production results.

[13] Stipkovits L., Lapis K., Hidvégi M., Kósa E., Glávits R., Resetár Á. (2004). Testing the efficacy of fermented wheat germ extract against Mycoplasma gallisepticum infection of chickens. *Poultry Science*.

[14] Ferenczi E. A. (2011). Fermentált búzacsíra kivonat hatása a broilerek Salmonella Infantis ürítésére, termelési mutatóira és egyes vakcinák által kiváltott szerológiai áthangolódásra.

[15] Mátis G. (2013). Effects of butyrate on hepatic epigenetics and microsomal drug-metabolizing enzymes in chicken.

[16] Mátis G., Kulcsár A., Petrilla J., Talapka P., Neogrády Z. (2016). Porcine hepatocyte-Kupffer cell co-culture as an in vitro model for testing the efficacy of anti-inflammatory substances. *Journal of Animal Physiology and Animal Nutrition*.

[17] Procházková D., Boušová I., Wilhelmová N. (2011). Antioxidant and prooxidant properties of flavonoids. *Fitoterapia*.

[18] Ishii T., Akagawa M., Naito Y. (2014). Pro-oxidant action of pyrroloquinoline quinone: characterization of protein oxidative modifications. *Bioscience, Biotechnology, and Biochemistry*.

[19] Sies H. (2017). Hydrogen peroxide as a central redox signaling molecule in physiological oxidative stress: oxidative eustress. *Redox Biology*.

[20] van der Vliet A., Janssen-Heininger Y. M. W. (2014). Hydrogen peroxide as a damage signal in tissue injury and inflammation: murderer, mediator, or messenger?. *Journal of Cellular Biochemistry*.

[21] El Kamouni S., El Kebbaj R., Andreoletti P. (2017). Protective effect of argan and olive oils against LPS-induced oxidative stress and inflammation in mice livers. *International Journal of Molecular Sciences*.

